# New Insights into Mn_1−x_Zn_x_Fe_2_O_4_ via Fabricating Magnetic Photocatalyst Material BiVO_4_/Mn_1−x_Zn_x_Fe_2_O_4_

**DOI:** 10.3390/ma11030335

**Published:** 2018-02-26

**Authors:** Taiping Xie, Chenglun Liu, Longjun Xu, Hui Li

**Affiliations:** 1 State Key Laboratory of Coal Mine Disaster Dynamics and Control, Chongqing University, Chongqing 400044, China; deartaiping@163.com (T.X.); xulj@cqu.edu.cn (L.X.); 2 Chongqing Key Laboratory of Extraordinary Bond Engineering and Advanced Materials Technology (EBEAM), Yangtze Normal University, Chongqing 408100, China; 3 College of Chemistry and Chemical Engineering, Chongqing University, Chongqing 400044, China; lihui@163.com

**Keywords:** magnetic photocatalyst, electron transfer, reaction kinetics, BiVO_4_, Mn-Zn ferrite, impregnation roasting method

## Abstract

BiVO_4_/Mn_1−x_Zn_x_Fe_2_O_4_ was prepared by the impregnation roasting method. XRD (X-ray Diffractometer) tests showed that the prepared BiVO_4_ is monoclinic crystal, and the introduction of Mn_1−x_Zn_x_Fe_2_O_4_ does not change the crystal structure of BiVO_4_. The introduction of a soft-magnetic material, Mn_1−x_Zn_x_Fe_2_O_4_, was beneficial to the composite photocatalyst’s separation from the liquid solution using an extra magnet after use. UV-vis spectra analysis indicated that Mn_1−x_Zn_x_Fe_2_O_4_ enhanced the absorption intensity of visible light for BiVO_4_. EIS (electrochemical impedance spectroscopy) investigation revealed that the introduction of Mn_1−x_Zn_x_Fe_2_O_4_ enhanced the conductivity of BiVO_4_, further decreasing its electron transfer impedance. The photocatalytic efficiency of BiVO_4_/Mn_1−x_Zn_x_Fe_2_O_4_ was higher than that of pure BiVO_4_. In other words, Mn_1−x_Zn_x_Fe_2_O_4_ could enhance the photocatalytic reaction rate.

## 1. Introduction

Photocatalysis and photocatalytic technology, which uses semiconductor materials to directly absorb and transmute renewable solar light energy into chemical energy, have been considered as promising methods to resolve environmental and energy problems facing the human population [1]. To date, cleaning up organic compounds via degradation and water splitting to produce H_2_ are the two most important applications of photocatalysis and its corresponding technology, which are aimed at environmental pollutant treatment and molecular hydrogen production, respectively [2].

In spite of the promising results regarding solar-light-driven photocatalysts, there are still some challenges inhibiting their practical application. The most pressing problem concerns the photocatalytic reaction kinetics using photocatalysts under solar light irradiation [3]. Most of the degradation reactions under solar light irradiation are very slow (they may take several hours). Therefore, it is very important to exploit new and highly efficient photocatalysts. In addition, the intrinsic relationship between photocatalytic activity and photocatalytic material structure can be elucidated by studying the photocatalytic mechanism, which will guide the synthesis and application of new and more efficient photocatalytic systems.

A monoclinic scheelite structure, BiVO_4_, with a better absorption ability of visible light, has attracted a great deal of attention due to its a relatively narrow band gap. The hybridization of Bi^6s^-O^2p^ orbitals upshifted the valence band of monoclinic BiVO_4_ to a lower potential at about +2.4 eV [2,4,5,6,7]. Nonetheless, the photocatalytic efficiency of BiVO_4_ is generally low because of its poor electron transfer and slow reaction kinetics.

Given that the photocatalyst materials could not be thoroughly recycled from a liquid solution after reaction, secondary pollution would probably be induced by the residual photocatalyst. This prevents it application as a wastewater treatment. Fortunately, a magnetic photocatalyst could solve the above issue [8,9]. The exploitation and application of various magnetic photocatalyst materials have been boosting scientists’ morale.

Here, we use Mn_1−x_Zn_x_Fe_2_O_4_ as a soft-magnetic substrate to prepare magnetic photocatalyst BiVO_4_/Mn_1−x_Zn_x_Fe_2_O_4_. In the composite system, the magnetic substrate, Mn_1−x_Zn_x_Fe_2_O_4_, facilitated the recovery of the photocatalyst from the liquid reaction solution using an extra magnet after the reaction. Most importantly, Mn_1−x_Zn_x_Fe_2_O_4_ could enhance the photocatalytic reaction rate of BiVO_4_ by enhancing the absorption intensity of visible light for BiVO_4_ and heightening the conductivity of BiVO_4_. We sincerely hope to extend the application field of Mn_1−x_Zn_x_Fe_2_O_4_ according to this report.

## 2. Experimental Procedure

All reagents purchased from Sinopharm Chemical Reagent Co., Ltd. (Shanghai, China) were of analytical grade purity and were used directly without further purification. The water used in all experimental processes was deionized water.

### 2.1. Preparation of BiVO_4_/Mn_1−x_Zn_x_Fe_2_O_4_

**Preparation of** **Mn_1_**_−_**_x_Zn_x_Fe_2_O_4_ [10]**. ZnSO_4_, MnSO_4_, and FeCl_3_⋅6H_2_O with a given molar ratio of n (ZnO): n (MnO): n (Fe_2_O_3_) = 13.3:32.8:53.9 was separately weighed and dissolved in water to form three solutions. Subsequently, the prepared ZnSO_4_ solution and FeCl_3_ solution were added into the MnSO_4_ solution under continuous stirring condition, in order to form the mixture solution. A definite amount of (NH_4_)_2_C_2_O_4_⋅H_2_O was dissolved to form a (NH_4_)_2_C_2_O_4_ solution. The prepared mixture solution was slowly added into the (NH_4_)_2_C_2_O_4_ solution, and the mixture solution and (NH_4_)_2_C_2_O_4_ solution were heated to 80 °C, respectively. The pH value of the whole reaction system was adjusted to 7 by slowly adding NaOH solution, and a large quantity of precipitate was observed. The precipitate was washed using water and dried at 80 °C for 12 h after filtrating to gain the precursor. The precursor was sintered at 1200 °C for 3 h to form the resultant magnetic substrate, Mn_x_Zn_1−x_Fe_2_O_4_.

**Preparation of BiVO_4_ [11]**. First, 0.01 mol Bi(NO_3_)_3_·5H_2_O and 0.02 mol tartaric acid were dissolved using a certain amount of 2 mol/L HNO_3_ solution to form a mixture solution A. Then, its pH value was adjusted to 7.5. Subsequently, 0.01 mol NH_4_VO_3_ was dissolved in 50 mL hot water (70 °C) to form solution B. Solution A and solution B were mixed to form the precursor of BiVO_4_. The precursor was dried at 80 °C for 24 h and then sintered at 500 °C for 4 h to obtain pure BiVO_4_.

**Preparation of BiVO_4_/Mn_1_**_−_**_x_Zn_x_Fe_2_O_4_**. First, 0.01 mol Bi(NO_3_)_3_·5H_2_O and 0.02 mol tartaric acid were dissolved with a certain amount of 2 mol/L HNO_3_ solution to form a mixture solution A. Then, its pH value was adjusted to 7.5. Subsequently, 0.01 mol NH_4_VO_3_ was dissolved in 50 mL hot water (70 °C) to from solution B. Solution A and solution B were mixed to form C solution. Then, 15.0 wt % Mn_x_Zn_1−x_Fe_2_O_4_ was added to solution C. BiVO_4_/Mn_x_Zn_1−x_Fe_2_O_4_ (15.0 wt %) was obtained after the mixture was dried at 80 °C for 24 h and then sintered at 500 °C for 4 h. The composites BiVO_4_/Mn_x_Zn_1−x_Fe_2_O_4_ (10.0%, 20.0%, 25.0 wt %) were prepared by adjusting the mass ratio of Mn_x_Zn_1−x_Fe_2_O_4_.

### 2.2. Materials Characterization

The structure characterization of the samples was determined by X-ray Diffractometer (Shimadzu, XRD-6000, Kyoto, Japan), Fourier transform infrared spectroscopy (FTIR, Perkin-Elmersystem 2000, Akron, OH, USA), and INVIA Raman microprobe (Renishaw Instruments, Wotton-under-Edge, UK). The light absorption properties, magnetization, and surface performances of products were examined by an ultraviolet-visible diffuse reflectance spectrophotometer (UV-vis, DRS, TU1901, Company, Beijing, China), vibrating sample magnetometer (VSM 7410, Lake Shore, Carson, CA, USA), Brunauer−Emmett−Teller (BET, ASAP-2020, Micromeritics, Norcross, GA, USA), and scanning electron microscopy (SEM, EVO-LS15X, ZEISS, Upper Cohen, Germany). The electrochemical workstation (PGSTAT30) was employed to measure the electrochemical impedance spectroscopy (EIS) of samples. The following are the test EIS parameters: K_3_[Fe(CN)_6_]:K_4_[Fe(CN)_6_] (1:1)-KCl electrolyte solution was employed. The work electrode content contained the prepared photocatalytic materials, acetylene black, and polytetrafluoroethylene (mass ratio, 85:10:5); the counter electrode was platinum foil; and the reference electrode was a saturated calomel electrode (SCE). Finally, the AC (Alternating Current) voltage amplitude was set at 5 mV and the frequency range was 1 × 10^5^~1 × 10^−2^ Hz.

### 2.3. Photocatalytic Tests

The photocatalytic activity of the prepared photocatalysts was investigated by the degradation of simulated dye wastewater (Rhodamine B, RhB) under visible light irradiation. One hundred milligrams photocatalyst was put into 100 mL RhB solution with a 5 mg/L concentration, then the suspension liquid was placed in the dark for 0.5 h with stirring to reach the adsorption-desorption equilibrium. A 500 W Xe lamp, equipping with a UV cut-off filter, was used as the visible light source (λ ≥ 420 nm). At the given irradiation time intervals, a series of the reaction solution was withdrawn and the absorbance was measured using the UV-vis spectrophotometer (TU-1901).

## 3. Results and Discussion

Primary analysis of photodegradation revealed that BiVO_4_/Mn_1−x_Zn_x_Fe_2_O_4_ (15 wt %) was the most efficient in the RhB degradation process under visible light irradiation.

### 3.1. Structure and Specific Surface Property

**Figure 1:**
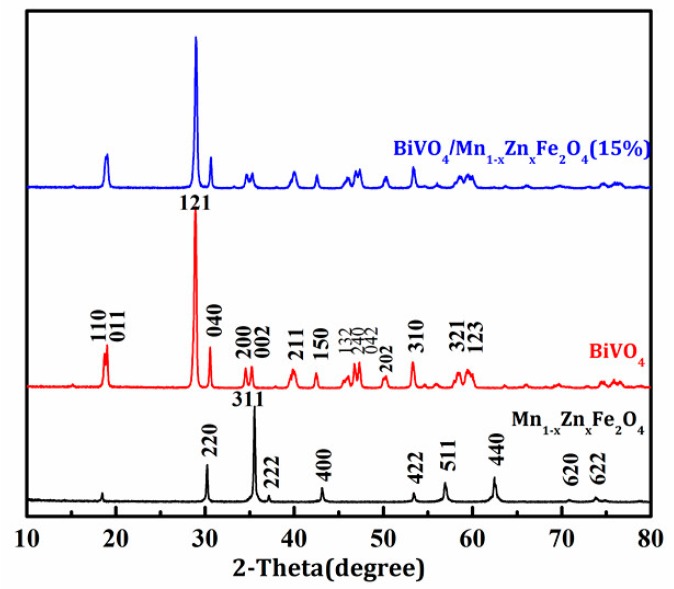
XRD (X-ray Diffractometer) patterns of the prepared samples.

Figure 1 shows the XRD patterns of the as-prepared samples. The characteristic spectra of monoclinic crystal BiVO_4_ was well indexed with the standard card (JCPDS file 14-0688), corresponding to the characteristic diffraction phases of (110), (011), (121), (040), (200), (002), (211), (150), (132), and (042) [12,13]. The diffraction pattern of Mn_1-x_Zn_x_Fe_2_O_4_ was fully matched with the standard card (JCPDS file 74-2400), with the characteristic reflection phases (220), (311), (222), (400), (422), (511), (440), (620), and (622) [10].

The diffraction peaks of the Mn_1−x_Zn_x_Fe_2_O_4_ pattern were difficult to observe in the pattern of BiVO_4_/Mn_1−x_Zn_x_Fe_2_O_4_ (15 wt %). One the one hand, the intensity of the diffraction peaks of Mn_1−x_Zn_x_Fe_2_O_4_ was relatively weak compared with that of the diffraction peaks of BiVO_4_. On the other hand, the diffraction patterns location of Mn_1−x_Zn_x_Fe_2_O_4_ overlapped with the domain diffraction patterns of BiVO_4_.

The peaks at (121) for BiVO_4_ in both patterns of pure BiVO_4_ and BiVO_4_/Mn_1−x_Zn_x_Fe_2_O_4_ (15 wt %) were clear, even in terms of their intensities. This phenomenon revealed that the introduction of Mn_1−x_Zn_x_Fe_2_O_4_ did not alter the growth orientation of BiVO_4_.

In addition, no impurity phases were found in the BiVO_4_/Mn_1−x_Zn_x_Fe_2_O_4_ (15 wt %) sample, confirming that there was no appreciable decomposition reaction for BiVO_4_ and Mn_1−x_Zn_x_Fe_2_O_4_ and no perceptible chemical reaction between the two components even though they were sintered at 500 °C.

**Figure 2:**
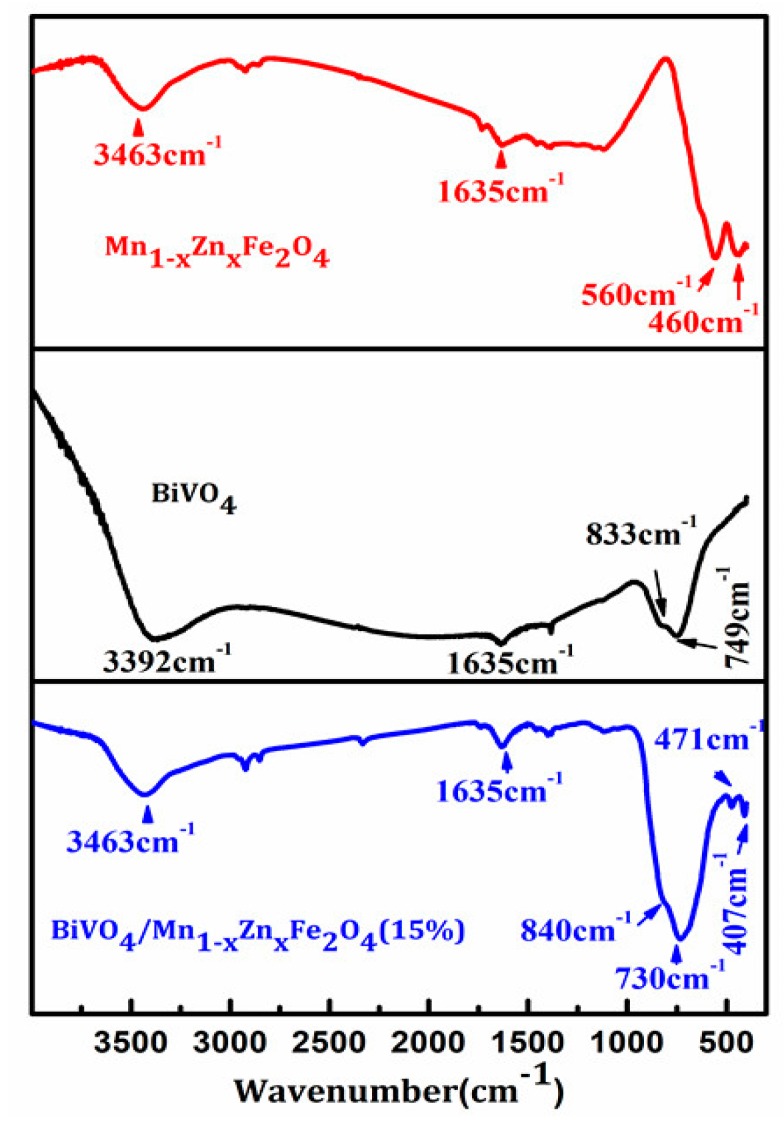
FTIR (Fourier transform infrared) spectrum of the prepared samples.

To further elucidate the structure of BiVO_4_/Mn_1−x_Zn_x_Fe_2_O_4_ (15 wt %), we carried out the measurement of Fourier transform infrared spectroscopy. Figure 2 illustrates the FTIR spectra of the as-prepared samples. The vibration absorption peaks of Mn-O, Zn-O, and Fe-O bands of Mn_1−x_Zn_x_Fe_2_O_4_ were at 560.1 cm^−1^, 473.7 cm^−1^, and 412.4 cm^−1^, respectively [14], while the V-O vibration absorption peaks of BiVO_4_ were at 734.3 cm^−1^ and 823.4 cm^−1^ [15]. This spectrum of BiVO_4_/Mn_1−x_Zn_x_Fe_2_O_4_ (15 wt %) could confirm the coexistence of Mn_1−x_Zn_x_Fe_2_O_4_ and BiVO_4_ in the prepared composite, which indicated that BiVO_4_/Mn_1−x_Zn_x_Fe_2_O_4_ (15 wt %) was prepared successfully. In addition, the absorption patterns at 2341.7 cm^−1^ and 3433.6 cm^−1^ were attributed to CO_2_ and the surface adsorption H_2_O [9].

**Figure 3:**
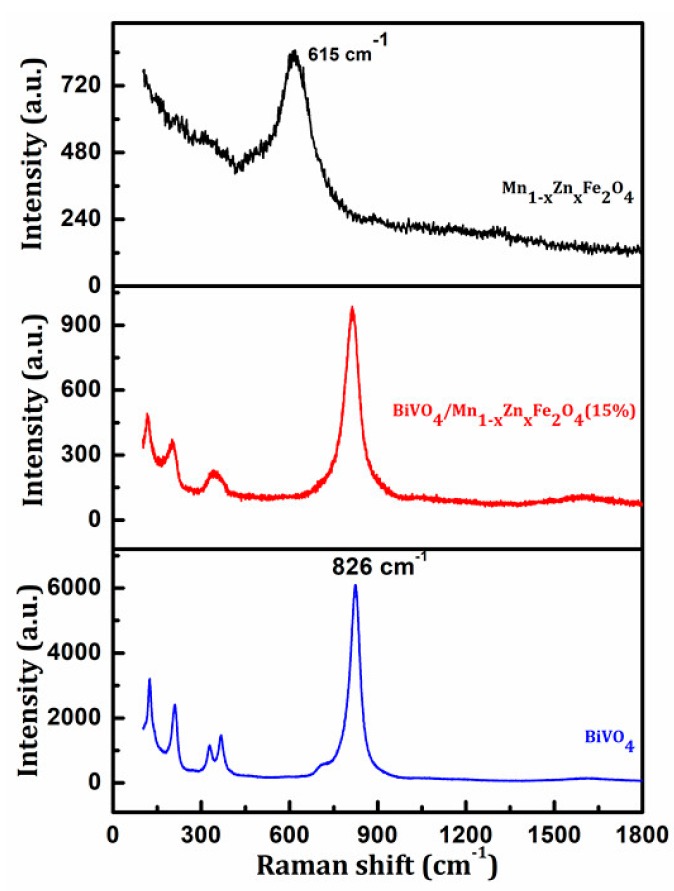
Raman spectra of the prepared samples.

Raman spectroscopy can provide structural information for materials, and is also a sensitive method to study the crystallization, local structure, and electronic properties of materials. The Raman spectra of the synthesized samples are shown in Figure 3. It can be seen from Figure 3 that the Raman band at 120 cm^−1^, 210 cm^−1^, 324 cm^−1^, 366 cm^−1^, and 826 cm^−1^ is the typical vibrational band of BiVO_4_ [16]. These bands could be also observed in the spectroscopy of BiVO_4_/ Mn_1−x_Zn_x_Fe_2_O_4_ (15 wt %), which further revealed that the BiVO_4_/Mn_1−x_Zn_x_Fe_2_O_4_ (15 wt %) was synthesized successfully, in agreement with the of XRD and FTIR analysis results. In addition, the intensity of BiVO_4_ was much larger than that of Mn_1−x_Zn_x_Fe_2_O_4_. So, Mn_1−x_Zn_x_Fe_2_O_4_ signal was not obvious in the spectra of BiVO_4_/Mn_1−x_Zn_x_Fe_2_O_4_ (15 wt %).

**Figure 4:**
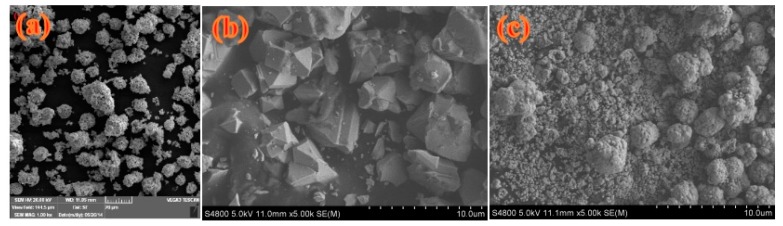
SEM (scanning electron microscopy) photographs of materials: (**a**) BiVO_4_, (**b**) Mn_1−x_Zn_x_Fe_2_O_4_, (**c**) BiVO_4_/Mn_1−x_Zn_x_Fe_2_O_4_ (15 wt %).

In order to further observe the morphology of the sample, we characterized the material using SEM. Figure 4 is the SEM diagram of the prepared sample: (a) BiVO_4_, (b) Mn_1−x_Zn_x_Fe_2_O_4_, and (c) BiVO_4_/Mn_1−x_Zn_x_Fe_2_O_4_ (15 wt %). It can be seen from Figure 4a that pure BiVO_4_ is a three-dimensional spherical structure, and Figure 4b shows that the prepared Mn_1−x_Zn_x_Fe_2_O_4_ is six-square-like structure. The larger sphere seen in Figure 4c is the core-shell structure of BiVO_4_ coated with Mn_1−x_Zn_x_Fe_2_O_4_, which indicated that the introduction of Mn_1−x_Zn_x_Fe_2_O_4_ caused the agglomeration of the resultant composite to some extent.

**Figure 5:**
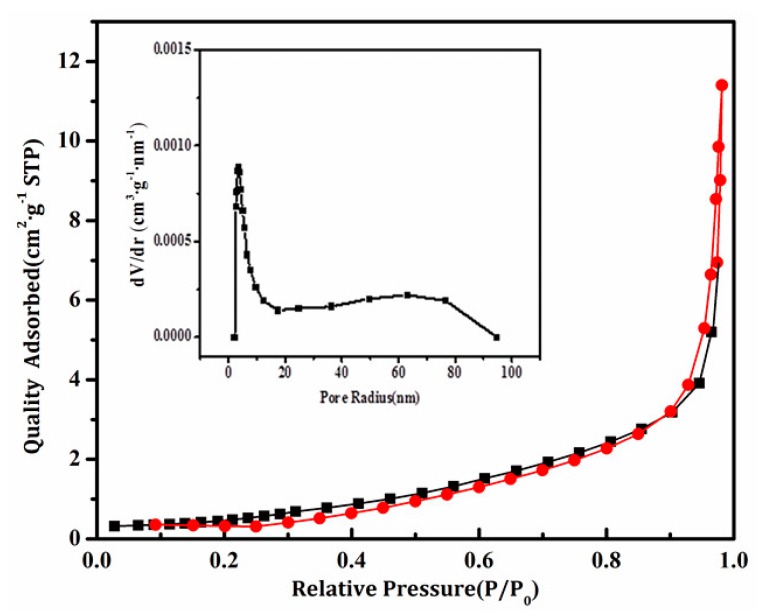
N_2_ adsorption-desorption isotherms and pore size distribution curves (insert) of BiVO_4_/Mn_1−x_Zn_x_Fe_2_O_4_ (15 wt %).

Figure 5 shows the N_2_ adsorption-desorption isotherm and the pore size distribution curve of BiVO_4_/Mn_1−x_Zn_x_Fe_2_O_4_ sample. The absorption-desorption isotherm could be categorized as a typical Type III absorption-desorption isotherm [17], indicating that the sample has a porous structure, which was convex to the P/P_0_ axis over its entire range, revealing that the as-prepared composite BiVO_4_/Mn_1−x_Zn_x_Fe_2_O_4_ (15 wt %) belonged to the nonporous structure. This sharp increase in the adsorption isotherm was attributed to the presence of macropores. The most probable pore size of BiVO_4_/Mn_1−x_Zn_x_Fe_2_O_4_ (15 wt %) is 6.0 nm. In addition, the specific surface area of BiVO_4_/Mn_1−x_Zn_x_Fe_2_O_4_ sample calculated by BET measurement is 2.22 m^2^/g.

### 3.2. Magnetic Properties

**Figure 6:**
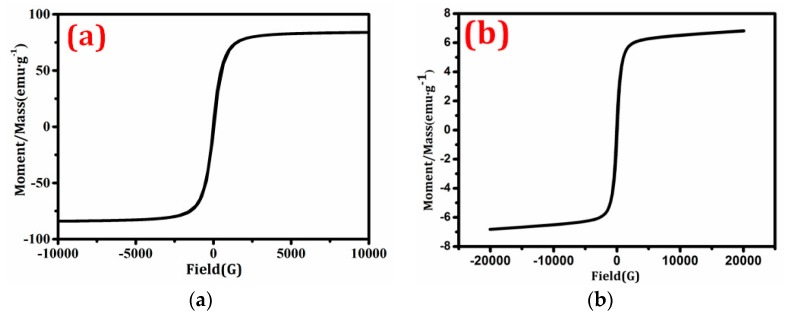
Hysteresis loops of (**a**) Mn_1−x_Zn_x_Fe_2_O_4_ and (**b**) BiVO_4_/Mn_1−x_Zn_x_Fe_2_O_4_ (15 wt %).

Figure 6 depicts the hysteresis loops of the magnetic substrate and magnetic photocatalyst BiVO_4_/Mn_1−x_Zn_x_Fe_2_O_4_ (15 wt %). The saturation magnetization (Ms) and remanent magnetization (Ms) of BiVO_4_/Mn_1−x_Zn_x_Fe_2_O_4_ (15 wt %) were 84.03 emu/g and 7.03 emu/g, respectively. Compared with pure Mn_1−x_Zn_x_Fe_2_O_4_, the Ms of the magnetic photocatalyst decreased by 75.4% and 91.6%, respectively, due to the reduction to the magnetic material content per unit mass of the magnetic photocatalyst [18]. Overall, the magnetic properties of BiVO_4_/Mn_1−x_Zn_x_Fe_2_O_4_ (15 wt %) were beneficial to its separation from the liquid solution and its recycling from the reaction solution using an extra magnet after use. In addition, the magnetic photocatalyst showed magnetic properties similar to pure Mn_1−x_Zn_x_Fe_2_O_4_, which indicated that the magnetic photocatalyst included Mn_1−x_Zn_x_Fe_2_O_4_.

### 3.3. UV-Vis DRS Analysis

**Figure 7:**
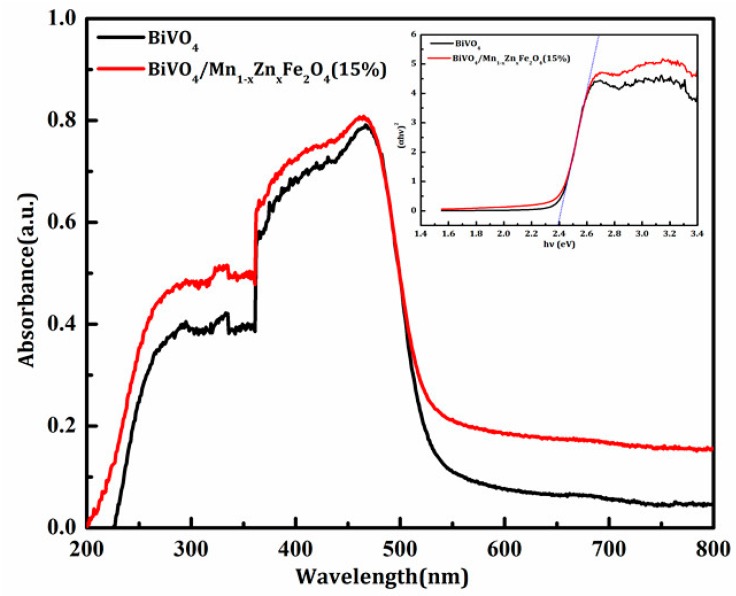
UV-Vis diffuse reflectance spectra of pure BiVO_4_ and BiVO_4_/Mn_1−x_Zn_x_Fe_2_O_4_ (15 wt %) samples. Inset: the plot of (Ah*v*)^2^ vs. hv to estimate the Eg value.

The optical absorption properties of semiconductors are considered to be a key factor affecting their photocatalytic activity. Figure 7 shows the UV-Vis diffuse reflectance spectra of BiVO_4_, BiVO_4_/Mn_1−x_Zn_x_Fe_2_O_4_, and the curve of (Ahv)^2^ vs. hv. It can be seen from the diagram that BiVO_4_ and BiVO_4_/Mn_1−x_Zn_x_Fe_2_O_4_ mainly absorb light in the wavelength range below 500 nm. Compared with pure BiVO_4_, the maximum absorption wavelength of BiVO_4_/Mn_1−x_Zn_x_Fe_2_O_4_ (lambda max) increases, and the absorption of visible light is enhanced to some extent. In addition, the band gap plays a very important role in the determination of photocatalytic activity. The relationship between absorbance and incident light intensity hv can be expressed by the following formula [8,9]:

Ah*v* = C (h*v* − Eg)^n/2^ (1)

In the above equation, A, Eg, h, and *v* represent the absorption coefficient, band gap width, Planck constant, and incident light frequency, respectively, and C is defined as a constant. The band gap energy of the sample can be obtained from the (Ah*v*)^2^~h*v* curve. The band gaps (Eg) of BiVO_4_ and BiVO_4_/Mn_1−x_Zn_x_Fe_2_O_4_ were estimated. They have nearly the same Eg values (2.36 eV), which are in agreement with the literature [5,19]. Though the incorporation of Mn_1−x_Zn_x_Fe_2_O_4_ could not extend the range of absorption light, it is worth noting that the anther function of Mn_1−x_Zn_x_Fe_2_O_4_ enhanced the absorption intensity of visible light for BiVO_4_ by charge transfer transitions (2Fe^3+^ → Fe^2+^ + Fe^4+^) [18].

### 3.4. Conductivity and Electrochemical Performance

**Figure 8:**
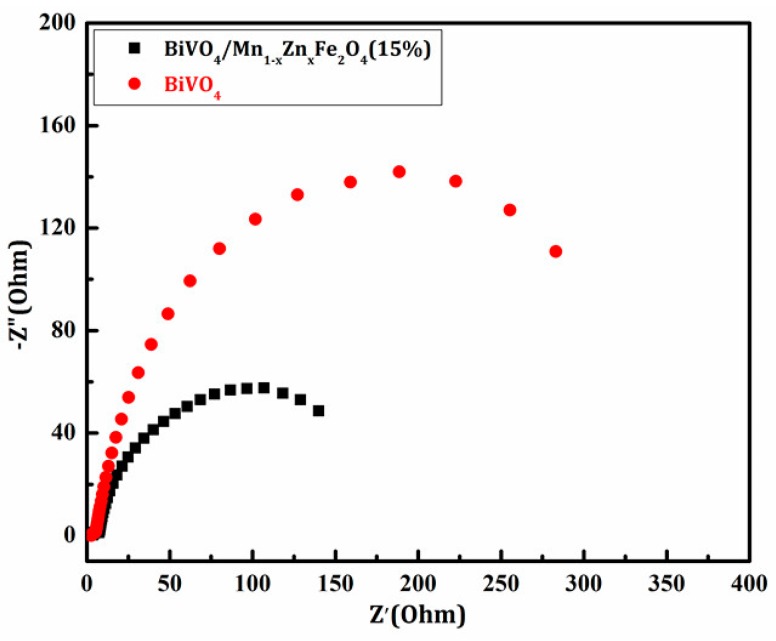
The Nyquist plots of BiVO_4_ and BiVO_4_/Mn_1−x_Zn_x_Fe_2_O_4_ (15 wt %).

Electrochemical impedance spectroscopy (EIS) is an effective method to evaluate the electron transfer between solid and electrolyte interfaces. Figure 8 shows the Nyquist diagram of the prepared samples. It can be seen from the diagram that the diameter of the semicircle decreases obviously with the introduction of Mn_1−x_Zn_x_Fe_2_O_4_, which indicates that the resistance of the solid interface layer and the surface electron transfer impedance decrease. The BiVO_4_/Mn_1−x_Zn_x_Fe_2_O_4_ charge transfer impedance (206 Ω·cm^2^) is less than pure BiVO_4_ (351 Ω·cm^2^) [20]. This is due to the doping effect of Fe ions in the Mn_1−x_Zn_x_Fe_2_O_4_ crystal lattice system. The charge transfer was easier, probably due to the charge transfer transitions of Fe ions (2Fe^3+^ → Fe^2+^ + Fe^4+^) and Mn ions (2Mn^4+^ → Mn^2+^ + Mn^6+^). The electron transfer impedance of the composite solid /electrolyte interface decreases, which may be because the incorporation of magnetic Mn_1−x_Zn_x_Fe_2_O_4_ could enhance the conductivity of BiVO_4_ and heighten the quantum efficiency of BiVO_4_. Thus, we can preliminarily predict that the photocatalytic activity of BiVO_4_/Mn_1−x_Zn_x_Fe_2_O_4_ must be higher than that of pure BiVO_4_ under the same light irradiation conditions.

### 3.5. Photocatalytic Activity and Stability

**Figure 9:**
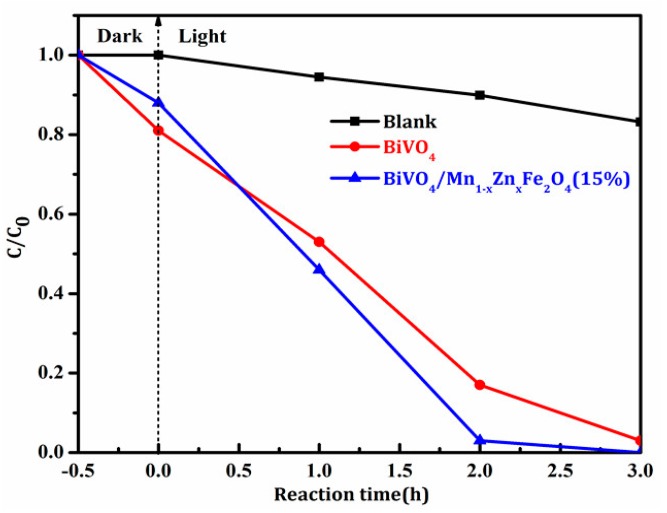
Photocatalytic degradation ratios of RhB with BiVO_4_ and BiVO_4_/Mn_1−x_Zn_x_Fe_2_O_4_ (15 wt %) samples.

The results of the RhB photodegradation by the prepared BiVO_4_ and BiVO_4_/Mn_1−x_Zn_x_Fe_2_O_4_ samples are shown in Figure 9. Obviously, the photocatalytic efficiency of BiVO_4_/Mn_1−x_Zn_x_Fe_2_O_4_ was higher than that of pure BiVO_4_. Under the same visible light irradiation, the degradation rate of RhB for pure BiVO_4_ reached 97% after 3 h, while BiVO_4_/Mn_1−x_Zn_x_Fe_2_O_4_ only took 2 h to achieve the same degradation rate. This result was in good agreement with the EIS analysis. On the one hand, the introduction of Mn_1−x_Zn_x_Fe_2_O_4_ enhanced the conductivity of BiVO_4_, further heightening the electron transfer ability. On the other hand, Mn_1−x_Zn_x_Fe_2_O_4_ enhanced the absorption intensity of visible light for BiVO_4_. Thus, BiVO_4_/Mn_1−x_Zn_x_Fe_2_O_4_ could produce more photoinduced electron-hole pairs under same visible light irradiation. The two factors generate a synergistic effect to boost the quantum efficiency of BiVO_4_.

**Figure 10:**
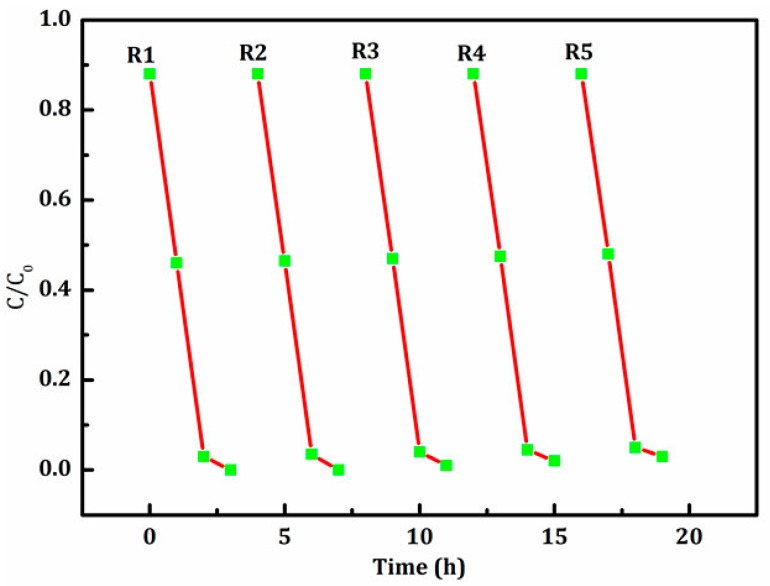
Recycling experiments of degrading RhB with BiVO_4_/Mn_1−x_Zn_x_Fe_2_O_4_ (15 wt %).

The repeatability of the magnetic photocatalyst was detected by cycling tests. After each cycle, the catalyst was separated by an external magnet, and then was washed and dried for the next cycle. The degradation rate of RhB using the fifth recycled photocatalyst was still more than 93% (see Figure 10). This indicated that the as-prepared magnetic photocatalyst had an excellent stability. In general, the introduction of a magnetic substrate can enhance the stability of a single component photocatalyst.

## 4. Conclusions

BiVO_4_/Mn_1−x_Zn_x_Fe_2_O_4_ was prepared by the impregnation roasting method. The synthesis method was suitable for the mass production of various composites. XRD tests showed that the prepared BiVO_4_ is monoclinic crystal, and the introduction of Mn_1−x_Zn_x_Fe_2_O_4_ does not change the crystal structure of BiVO_4_. The introduction of a soft-magnetic material, Mn_1−x_Zn_x_Fe_2_O_4_, was beneficial to the composite photocatalyst’s separation from the liquid solution using an extra magnet after the reaction. UV-vis spectra analysis indicated that Mn_1−x_Zn_x_Fe_2_O_4_ enhanced the absorption intensity of visible light for BiVO_4_. EIS investigation revealed that the introduction of Mn_1−x_Zn_x_Fe_2_O_4_ could decrease the electron transfer impedance of BiVO_4_, further enhancing its conductivity and heightening its quantum efficiency. The photocatalytic efficiency of BiVO_4_/Mn_1−x_Zn_x_Fe_2_O_4_ was higher than that of pure BiVO_4_.
